# Effectiveness of RHZE-FDC (fixed-dose combination) compared to RH-FDC + Z for tuberculosis treatment in Brazil: a cohort study

**DOI:** 10.1186/s12879-015-0820-4

**Published:** 2015-02-21

**Authors:** José Ueleres Braga, Anete Trajman

**Affiliations:** National School of Public Health, Fiocruz, Rio de Janeiro, Brazil; Social Medicine Institute, Rio de Janeiro State University, Rio de Janeiro, Brazil; Federal University of Rio de Janeiro, Rio de Janeiro, Brazil; Montreal Chest Institute, McGill University, Montreal, Canada

**Keywords:** Effectiveness, Fixed-dose combination, Treatment, Tuberculosis

## Abstract

**Background:**

In 2009, Brazil was the sole high-burden country to use three drugs [rifampin (R), isoniazid (H) and pyrazinamide (Z)] as the standard treatment for sensitive tuberculosis, with RH in fixed-dose combination (FDC). In December 2009, the country has adopted the FDC four-drug regimen including ethambutol (E). The rationale was the expectation to reduce default and resistance rates, by increasing adherence to treatment and avoiding monotherapy. However, there is no consensus on the superior effectiveness of the RHZE-FDC regimen over RH-FDC + Z. In particular, few studies evaluated its influence on default and smear negativation rates.

**Methods:**

We conducted a historic cohort study to assess the effectiveness of RHZE-FDC for the treatment of tuberculosis in Brazil, measured by the rates of treatment default and smear negativation in the second month of treatment, using secondary data from the national information system known as SINAN-TB.

**Results:**

The RHZE-FDC had a protective effect against treatment default compared to RH-FDC + Z, reducing it by 14%. However, it was not possible to show an effect of the RHZE-FDC on the rate of second month smear negativation. In addition to the regimen, other well-studied individual characteristics, such as older age (over 38 years) and higher education occupation were also protective against default. Conversely, alcoholism increased the probability of defaulting. These programmatic findings suggests the benefits of RHZE-FDC over RH-FDC + Z.

**Conclusion:**

Our analysis of a cohort database in a high burden country shows that compared to RH-FDC + Z, RHZE-FDC reduces the default rates, independently of other influencing individual or health service factors.

**Electronic supplementary material:**

The online version of this article (doi:10.1186/s12879-015-0820-4) contains supplementary material, which is available to authorized users.

## Background

Tuberculosis is a public health problem in Brazil. The country is part of the list of the 22 high-burden countries since its first edition [1], despite the recent advances in the control of the epidemics: incidence rates decreased from 47 in 2001 to 36 new cases/100,000 inhabitants in 2012. Nevertheless, cure (79%) and default (12%) rates remain way beyond those needed to attain the millennium development goals up to 2050 [2], to which the Brazilian Ministry of Health (MoH) committed [3].

The advances in tuberculosis control, despite insufficient, were obtained after the adoption of a few strategies by the MoH [4]. Treatment for tuberculosis is free of charge in the country and includes the short regimen with rifampin since the seventies. Since the eighties, the MoH has strengthened the primary care services, and since the nineties, adopted the Family Health Strategy [5]. Additionally, the directly observed therapy (DOT) was recommended nationally [6].

However, to face the challenge of further improvement of operational tuberculosis indicators, new strategies were proposed. In the end of 2009, in the hope of reducing the rates of treatment default and halting the possible increase in resistance rates, the Brazilian National Tuberculosis Program (NTP) recommended the adoption of the World Health Organization (WHO)-recommended regimen: Rifampin (R), Isoniazid (H), Pyrazinamide (Z) and Ethambutol (E) in fixed-combined doses (_2_RHZE-FDC/_4_RH-FDC) as replacement for the _2_RH-FDC + Z/_4_RH-FDC in use at that time. This change was recommended for adults and adolescents over 10 years [7]. It was expected that the FDC presentation would increase treatment effectiveness by reducing default because of the smaller number of pills to be swallowed and because of possible better tolerance, since standard doses of H and Z for those weighting over 50 kg were reduced from 400 to 300 mg and from 2000 to 1200 mg, respectively [8]. Additionally, the NTP expected to halt the increase in resistance because the possibility of monotherapy would be avoided. Moreover, the addition of E was expected to decrease failure among patients with suspected H-resistant TB. Despite the alarming increase in multidrug-resistant tuberculosis in many parts of the world, resistant rates remain low in Brazil. Finally, an easier administration of drug supply was expected with the “new” regimen.

However, there is no consensus in the literature on the higher efficacy of the FDC regimen when compared to separate drug regimens for treating tuberculosis. The higher efficacy of the RHZE over RHZ is also poorly studied, mainly in countries having adopted it recently. Three systematic reviews (SR) on FDC versus separate drug regimens for tuberculosis treatment are available [9-11]. In the recently published (2013) SR by Albanna et al. [9] that included 15 randomized control trials (RCT), FDC was not associated with reduction in failure, relapse, two-month smear/culture positivity (an early surrogate for cure), or emergence of drug resistance as compared to separate drug formulation. Out of five RCTs that evaluated adherence, none favoured FDC formulations.

Monedero & Caminero [11] also concluded that regarding cure and relapse rates, the FDC are not superior to separate drugs for treating tuberculosis. Only one out of the 15 included studies evaluated prevention of resistance, and other important outcomes, such as default rates and two-month smear negativation, were not evaluated in these studies. The authors concluded that the FDC regimen should be recommended for logistic reasons, costs and practicability, but not on effectiveness grounds.

The third SR, dated 2004, is a more generic review of FDC treatments and included two studies on tuberculosis [10], which did not corroborate the superiority of this regimen.

Thus, little information is available on the effectiveness of the RHZE-FDC in high burden countries that have already adopted other strategies, such as DOT and decentralization for tuberculosis control. The present study aimed to evaluate the effectiveness of RHZE-FDC compared to RH-FDC + Z for tuberculosis treatment in Brazil, using a historical cohort database with secondary data from the national surveillance information system (*Sistema de Informação de Agravos de Notificação da Tuberculose* – SINAN-TB). Outcomes were treatment default and negativation of smears at the second month of treatment.

## Methods

We conducted a non-concurrent cohort observational study to evaluate the effects of interventions applied to subjects diagnosed and treated in Brazilian health care units from five cities. In Brazil, tuberculosis is a compulsorily notified disease and both diagnosis and treatment are provided free of charge in the public national health system (*Sistema Único de Saúde*-SUS). There are no individual selection criteria for the use of any tuberculosis treatment regimen in new cases in Brazil. The NTP distributes the drugs to the State and Municipal Health Departments, which distribute to health units upon case notification. The adoption of FDC was a programmatic decision and its implementation was progressive, during our study period. Once the health unit implements the regimen, all patients will receive the same treatment. Other regimens will only be available for intolerance or resistance.

We selected new tuberculosis cases (untreated previously) who started treatment from October 2009 to September 2010, residents in five eligible cities according to the following criteria: (*i*) cities in the five geographical regions of the country; (*ii*) with at least 120 notified cases yearly; (*iii*) with different incidence rates (over and above the national incidence rate) e (*iv*) with different proportions of cases using RHZE-FDC (under 30%, between 30 and 70% and over 70%) during the study period.

Based on these criteria, the following cities were selected by convenience: Goiânia (Central region, low incidence), Manaus, Salvador and Porto Alegre (high incidence, over 70% use of FDC-RHZE in the North, Northeast and South regions, respectively) and Rio de Janeiro (high incidence, under 30% of FDC-RHZE use, Southeast region).

Since besides individual characteristics, health service-related characteristics can influence the outcomes of this kind of intervention, both were collected and analysed as independent variables in a hierarchized model. Individual variables present in SINAN and included in our analyses were: (*a*) sex, (*b*) age group, (*c*) schooling, (*d*) occupation, (*e*) living in prisons, (*f*) alcoholism, (*g*) diabetes, (*h*) mental disease, (*i*) HIV infection, (*j*) other co-morbidities and (*k*) DOT. Health care service-related variables were: (1) facilities treating more than 125 cases yearly, (2) DOT implemented in the unit, (3) follow-up smears requested in the unit, (4) units having their own laboratorial facilities, (5) complete health team, including physician, nurse and social worker, (6) units with a social service, (7) units with community health care agents, (8) units with the Family Health Strategy, (9) units with training activities, (10) units with primary care services (11) units having exclusively primary care. These facility-related data were extracted from the National Registry of Health Care Units (*Cadastro Nacional de Estabelecimentos de Saúde*), available at *cnes.datasus.gov.br*, accessed on July 12 2012, and validated with the municipal tuberculosis program by one of the authors.

The notification system allows entry of separate drugs used for the treatment of tuberculosis. Until the adoption of the FDC-RHZE treatment, E was not used in the country for new cases, only for retreatment cases. Hence, we considered that reported new cases using E after the implementation of RHZE-FDC in the city were using the FDC formulation. Likewise, registries of new cases using R, H and Z only were considered to be using the previous RH-FDC + Z regimen.

The effectiveness was evaluated using two outcomes: default (according to the NTP guidelines, 30 days without treatment or 60 days without follow up) and negativation of smear at the second month of treatment (a hallmark for deciding on treatment change according to the National Guidelines) [8]. Both treatment outcomes were obtained in the follow-up SINAN database. Details on the quality of the SINAN database during the study period are available elsewhere [12]. In summary, a previous quality evaluation including completeness and consistence criteria had shown that the database was suitable for this kind of analysis.

A hierarchized logistic regression model was used considering two levels, the individual and the collective. The final model originated from the specific models for each of both levels. A significance cut-off of 0.2 was considered to include variables from the specific level models. Using the stepwise method, the final model considered a 0.05 significance cut-off.

The use of the tuberculosis surveillance database was authorized by the Brazilian NTP under confidentiality condition. The study was approved by the Institutional Review Board of the Social Medicine Institute of Rio de Janeiro State University and by the National Ethical Committee (25000.219561/2011-72). Since this study was based on secondary data, routinely collected and registered in the national surveillance information system, informed consent was not obtained from individual patients. The ethical boards were aware and exempted from the consent procedure. The intervention (treatment with RHZE-FDC) was not the researchers decision, we just documented the differences between patients, services and cities having implemented the recommended regimen or not.

## Results

A total of 11,111 patients were included, of whom 46% were residents in Rio de Janeiro, situated in the Southeast region, which concentrates more than half the Brazilian population; 23% in Salvador, 14% in Manaus and Porto Alegre each and 3% in Goiânia. Table 1 shows the distribution of patients in the cities according to the type of treatment.

**Table 1:** **Distribution of pulmonary tuberculosis patients per city according to the type of treatment from October 2009 to September 2010**

**City**	**FDC-RHZE**		**RH-FDC + Z**		**Total**	
	**N**	**%**	**N**	**%**	**N**	**%**
Goiania	120	41.5	169	58.5	289	100.0
Manaus	1181	75.1	391	24.9	1572	100.0
Porto Alegre	859	54.3	724	45.7	1583	100.0
Rio de Janeiro	205	4.0	4942	96.0	5147	100.0
Salvador	1372	54.4	1148	45.6	2520	100.0
Total	3737	33.6	7374	66.4	11111	100.0

FDC = fixed-dose combination; R = rifampin; H = isoniazid; Z = pyrazinamide; E = ethambutol.

Patients who received the RHZE-FDC regimen were more likely to be older, illiterate, alcoholics, have other co-morbidities considering a 0.05 significance level; while those under RH-FDC + Z were more likely to be in prisons (Table 2). These differences indicated that patients’ heterogeneity should be taken into account in the evaluation of the effect of the intervention on the outcomes. They could be also due to differences overtime, since RHZE-FDC patients corresponded to more recent notifications in the same city.

**Table 2:** **Characteristics of pulmonary tuberculosis patients according to the type of treatment from October 2009 to September 2010**

**Characteristics**	**FDC-RHZE**		**RH-FDC + Z**		**Total**		***p*** **-value**
	**N**	**%**	**N**	**%**	**N**	**%**	
Age							
<38 years	1952	52.2	4100	55.6	6052	54.5	0.001
≥38 years	1785	47.8	3.274	44.4	5059	45.5	
Total	3737		7374		11111		
Sex							
Feminine	1301	34.8	2539	34.4	384	34.6	0.689
Masculine	2436	65.2	4835	65.6	7271	65.4	
Total	3737		7374		11111		
Illiteracy							
No	3487	96.4	7035	97.4	10522	97.0	0.003
Yes	132	3.7	189	2.6	321	3.0	
Total	3619		7224		10843		
Higher education occupation							
No	2773	97.0	5488	96.6	8261	96.7	0.384
Yes	87	3.0	193	3.4	280	3.3	
Total	286		5681		8541		
Living in prisons							
No	3362	91.8	6320	87.9	9682	89.2	0.000
Yes	302	8.2	869	12.1	1171	10.8	
Total	3664		7189		10853		
Alcohol use							
No	2835	81.8	5256	85.8	8091	84.3	0.000
Yes	632	18.2	870	14.2	1502	15.7	
Total	3467		6126		9593		
Diabetes							
No	3174	92.4	5505	91.6	8679	91.9	0.174
Yes	262	7.6	505	8.4	767	8.1	
Total	3436		6010		9446		
Mental disorder							
No	3363	97.8	5896	97.4	9259	97.5	0.194
Yes	76	2.2	160	2.6	236	2.5	
Total	3439		6056		9495		
Other co-morbidities							
No	2640	81.3	4687	84.0	7327	83.0	0.001
Yes	608	18.7	896	16.1	1504	17.0	
Total	3248		5583		8831		
DOT							
No	2746	75.9	5390	76.0	8136	75.9	0.954
Yes	872	24.1	1706	24.0	2578	24.1	
Total	3618		7096		10714		
HIV status							
Negative	1267	74.7	2011	76.7	3278	75.9	0.135
Positive	430	25.3	614	23.4	1044	24.1	
Total	1697		2625		4322		

FDC = fixed-dose combination; R = rifampin; H = isoniazid; Z = pyrazinamide; E = ethambutol, DOT = directly observed treatment.

Regarding health service characteristics, patients receiving RHZE-FDC were more likely to be treated in units treating less than 125 cases per year, with DOT implemented, performing follow-up smears and having exclusively primary care services. Conversely, those receiving RH-FDC + Z were more likely to have complete health teams, a social service, community health agents, the Family Health Strategy, teaching activities and primary care (Table 3).

**Table 3:** **Characteristics of health care units where pulmonary tuberculosis patients were treated according to the type of treatment from October 2009 to September 2010**

**Characteristics**	**FDC-RHZE**		**RH-FDC + Z**		**Total**		***p*** **-value**
	**N**	**%**	**N**	**%**	**N**	**%**	
The unit assists over 125 cases yearly							
No	1989	53.2	3574	48.5	5563	50.1	0.000
Yes	1748	46.8	3800	51.5	5548	49.9	
Total	3737		7374		11111		
The unit offers DOT							
No	440	11.8	1621	22.0	2061	18.6	0.000
Yes	3297	88.2	5753	78.0	905	81.5	
Total	3737		7374		11111		
The unit requests follow up smears							
No	283	7.6	2135	29.0	2418	21.8	0.000
Yes	3454	92.4	5239	71.1	8693	78.2	
Total	3737		7374		11111		
The unit has a laboratory facility							
No	2477	66.3	4763	64.6	7240	65.2	0.070
Yes	1260	33.7	2611	35.4	3871	34.8	
Total	3737		7374		11111		
The unit has a complete team with physician, nurse and social worker							
No	2153	57.6	1872	25.4	4025	36.2	0.000
Yes	1584	42.4	5502	74.6	7086	63.8	
Total	3737		7374		11111		
The unit has a Social Service							
No	2153	57.6	1872	25.4	4025	36.2	0.000
Yes	1584	42.4	5502	74.6	7086	63.8	
Total	3737		7374		11111		
The unit has community health agents							
No	2626	70.3	2980	40.4	5606	50.5	0.000
Yes	1111	29.7	4394	59.6	5505	49.6	
Total	3737		7374		11111		
The unit has the Family Health Program							
No	2664	71.3	3573	48.5	6237	56.1	0.000
Yes	1073	28.7	3801	51.6	4874	43.9	
Total	3737		7374		11111		
The unit has training activities							
No	3534	94.6	6249	84.7	9783	88.1	0.000
Yes	203	5.4	1125	15.3	1328	12.0	
Total	3737		7374		11111		
The unit has primary care							
No	2692	72.0	2205	29.9	4897	44.1	0.000
Yes	1045	28.0	5169	70.1	6214	55.9	
Total	3737		7374		11111		
The unit has exclusively primary care							
No	2832	75.8	6543	88.7	9375	84.4	0.000
Yes	905	24.2	831	11.3	1736	15.6	
Total	3737		7374		11111		

FDC = fixed-dose combination; R = rifampin; H = isoniazid; Z = pyrazinamide; E = ethambutol, DOT = directly observed treatment.

### The effect of FDC-RHZE on treatment default

In the present study period, in the five cities, default was the treatment outcome in 14% of new cases. This rate varied from 7.5% in Salvador to 20.3% in Porto Alegre. The FDC-RHZE regimen appears to have a protective effect against default compared to the RH-FDC + Z regimen but this lost significance when adjusted for individual characteristics. Older age and higher education occupation had a protective effect while alcoholism and HIV co-infection were directly associated to defaulting (Additional file 1: Table S1).

When health clinic characteristics are considered, the FDC-RHZE regimen has a protective effect, even when adjusted for the characteristics that influence default. The existence of a laboratory facility in the health unit was the most important characteristic protecting against default. Other protective characteristics were DOT adoption and follow-up smears (Additional file 1: Table S2).

The final hierarchized model including both individual and health facility characteristics showed that the FDC-RHZE has a 14% protective effect against default, detectable when excluded the influence of the other aspects that are also associated with default (Table 4).

**Table 4:** **Risk factors for TREATMENT DEFAULT among pulmonary tuberculosis patients from October 2009 to September 2010 (hierarchized model)**

**Characteristics**	**%**	**OR**	**95% CI**	_adj_ **OR**	**95% CI**
FDC-RHZE					
No	66.4	1		1	
Yes	33.6	0.811	0.723 - 0.910	**0.862**	**0.523 - 0.998**
Age					
<38 years	54.4	1		**1**	
≥38 years	45.5	0.620	0.555 - 0.692	**0.599**	**0.484 - 0.742**
Higher education occupation					
No	97.0	1		**1**	
Yes	3.0	0.355	0.217 - 0.582	**0.223**	**0.081 - 0.610**
Alcohol use					
No	81.8	1		**1**	
Yes	18.2	1.555	1.349 - 1.793	**1.767**	**1.377 - 2.268**
The unit offers DOT					
No	18.5	1		1	
Yes	81.5	0.946	0.826 - 1.082	0.979	0.850 - 1.128
The unit requests follow up smears					
No	21.7	1		1	
Yes	78.3	0.914	0.805 - 1.037	0.947	0.826 - 1.087
The unit has a laboratory facility					
No	65.2	1		1	
Yes	34.8	0.904	0.808 - 1.012	**0.892**	**0.796 - 1.001**

Bold numbers are statistically significant findings. FDC = fixed-dose combination; R = rifampin; H = isoniazid; Z = pyrazinamide; E = ethambutol, DOT = directly observed treatment; OR = odds ratio; _adj_OR = adjusted odds ration; CI = confidence interval.

### The effect of FDC-RHZE on negativation of second month follow-up smears

The information on follow up smear at the second month was available for 2,792 (25.1%) patients. Negativation of second month smear occurred in 68% of them. The highest rate of negativation was observed in Manaus (74%) and the lowest in Rio de Janeiro (56%).

Compared to those using RH-FDC + Z, patients receiving FDC-RHZE had a higher probability of having a negative smear on the second month of treatment. The individual characteristics influence little this outcome, a part from a using DOT (Additional file 1: Table S3).

However, when simultaneously considering the health facility-related characteristics, the protection by FDC-RHZE is not sustained. Patients treated in units with more than 125 cases yearly are less likely to have a negative smear on the second month, while the inverse is observed among those treated in units that adopted DOT or have exclusively primary care services (Additional file 1: Table S4).

The final, hierarchized model shows that FDC-RHZE has no influence on the second month-smear negativation. The other variables with an influence in the specific model sustained significance in the final model (Table 5).

**Table 5:** **Risk factors for SECOND MONTH SMEAR NEGATIVATION among pulmonary tuberculosis patients from October 2009 to September 2010 (hierarchized model)**

**Characteristics**	**%**	**OR**	**95%** **CI**	_adj_ **OR**	**95%** **CI**
FDC-RHZE					
No	66.4	1		1	
Yes	33.6	1.104	0.932 - 1.307	1.164	0.999 - 1.372
DOT					
No	75.9	1		1	
Yes	24.1	1.450	1.208 - 1.739	**1.373**	**1.137 - 1.658**
The unit assists over 125 cases yearly					
No	50.1	1		1	
Yes	49.9	0.782	0.663 - 0.922	**0.817**	**0.686 - 0.974**
The unit has exclusively primary care					
No	84.4	1		1	
Yes	15.6	1.289	1.065 - 1.559	**1.336**	**1.099 - 2.126**

Bold numbers are statistically significant findings. FDC = fixed-dose combination; R = rifampin; H = isoniazid; Z = pyrazinamide; E = ethambutol, DOT = directly observed treatment; OR = odds ratio; _adj_OR = adjusted odds ration; CI = confidence interval.

## Discussion

In the present study, default rate was high (14%), significantly higher than the rate tolerated by WHO (5%) [13]. This is not a surprise, given the default rates reported in the country as a whole [3]. Our analysis showed that as compared to RH-FDC + Z, RHZE-FDC had a protective effect of 14% against defaulting from tuberculosis treatment. However, no protective effect was observed for second month-smear negativation.

Studies comparing FDC and separated drug outcomes using secondary notification data were not found in the literature. However, the set of controlled trials in the last two decades that analysed FDC versus single drugs for tuberculosis treatment found no protective effects on cure, relapse or smear negativation rates [11]. However, programmatic, operational studies can have different outcomes when compared with the controlled conditions of a randomized controlled trial. In our evaluation using the MoH database, previously shown to be suitable for this type of analyses, we did not find such protection either, at least when compared to RH-FDC + Z. The outcome default using FDCs has been less explored in the literature. Both older (1987) [14] and more recent (2002) [15] studies did not detect any difference in default rates, although smear negativation rates increased with FDC in the former, as well as in more recent studies [16].

The most important experimental study on this matter was recently published by Lienhardt et al. [17]. This randomized controlled trial known as *The Study C* was conducted in 11 sites in Africa, Asia and Latin America between 2003 and 2008. Unlike ours, this study aimed to evaluate safety and effectiveness of FDC-RHZE compared to RHZE in separate formulation, and the main outcome was a negative culture at 18 months after treatment onset. The study concluded that the non-inferiority condition was attained and that FDCs are better accepted because of the potential advantages associated with their administration as compared to the separate drugs. Unlike the previously reported studies, ours uses an observational methodology, and two different regimens (not only the formulation) are compared. Thus, we cannot conclude that the benefits of the RHZE-FDC regimen are due exclusively to its fixed-dose presentation, since doses were different and an additional drug (E) was included.

Because randomization is not possible in the used study design, we adjusted for potential confounding variables, both at the individual and at the health service level. Our results indicate that at the individual level, besides the regimen, patients with older age and higher education occupation, a proxy for socioeconomic status, were less likely to default, while alcoholism increased the risk for default. These individual variables were largely explored in the literature and there is solid evidence to support their role in defaulting, as summarized in a meta-analysis [18,19]. Other characteristics not present in SINAN database, such as adverse events and unemployment, and other not amenable in this king of study, such as knowledge and believes, have also been shown to influence default rates [18,19] It is beyond of the scope of the present study to discuss each of these factors. They were only analysed to verify if the effect of the RHZE-FDC regimen was spurious or independent of these variables, since there was no randomization. Likewise, although drug bioavailability is another important factor explored in FDC studies, this discussion is out of the scope of the present analysis [20,21]. Finally, regarding the laboratory outcome, negative culture at 18 months, recommended as an endpoint by international bodies to evaluate failure and relapse, was not available in the Brazilian surveillance database. Second-month negativation is indeed, not a sensitive surrogate for these outcomes. Our choice of the second-month negativation as an outcome was however justified because [1] it was the best available information, since it is a national recommendation to request culture if the mandatory sputum smear at the second month is positive and [2] the rationale for the regimen replacement was high rates of default and of resistance, and the second-month negativation is a good predictor of resistance.

Adherence is probably the factor that most influence the effectiveness of any treatment [22]. This is the reason why we chose default as one of the outcomes in the present analysis. Besides influencing effectiveness, irregular and incomplete tuberculosis treatments are also responsible for emergence of multidrug resistant-tuberculosis (which we could not evaluate).

Interestingly, while only individual characteristics and the FDC regimen had an effect on default, only health care characteristics, such as DOT, service organization and workload influenced the smear negativation rate. The Family Health Strategy increases the bonding between the community and the health services [5]. This attachment, mostly developed through the community health agent, may be at the origin of better tuberculosis treatment outcomes in Brazil [19,23]. Likewise, despite the controversy [24,25], DOT has been recognized as an excellent strategy that has prevented 4.6–6.3 million deaths between 1995 and 2009 in the world since its implementation [26]. Finally, the importance of a multidisciplinary team to deal with this socially determined disease could not be overemphasized.

Individual characteristics presented by patients from our sample might have been a result of selection bias. However, they had the same profile as those not included in the analyses (data not shown) thus we do not believe our results are a consequence of bias. Another possible limitation would be the quality of data itself, since the source of the database was the routine surveillance information system. Nevertheless, a previous quality control [12] showed that the data gathered was adequate for analysis. The sputum negativation outcome was available only for one quarter of patients, reducing the statistical power for this outcome. However, it is unlikely that it jeopardizes the validity of the results. In addition, despite selecting new cases, multiresistant cases could have been included, but again, it is unlikely that those patients could have selectively received one of the treatment regimens. Finally, a classification bias might have occurred because we considered any RHZE as using FDC formulation in the period of the intervention. Although this can have overestimated the number of patients under FDC, it is unlikely that it had any effect on the analysis of individual and collective factors associated with the selected outcomes.

On the other hand, the inclusion of several cities with heterogeneous health unit performance and different probabilities of use of the treatment regimens allowed a wide spectrum of individual and health system characteristics, necessary for adjustments of the effect measures. For example, most patients in Rio received the RH-FDC + Z regimen in heterogeneous health units. This is the reason why we adjusted for health unit characteristics and not for cities. In addition, the option of conducting a study with an internal comparison group instead of a “before and after” design reduces the chances of observing effects (such as default rates) that could be attributed to other non-controlled interventions concurrent to the implementation of the new regimen.

Although observational studies are not ideal for evaluating programmatic interventions, this was the only possible method because the intervention was already launched when we planned the study. On the other hand, it had the advantage of showing the pragmatic effect of the “new” treatment on notified cases. Retrospectively, it strengthens the Brazilian NTP decision to change the treatment in the country. As new, shorter regimens are under investigation and expected for a near future (as pointed at http://www.tballiance.org/downloads/Pipeline/TBA%20Pipeline%20Q1%202015%282%29.pdf), it is important to plan cluster randomized studies to evaluate the effectiveness of the intervention at the health service level. However, secondary data from the national surveillance system is also a source of reliable and useful information in early adopter countries.

## Conclusions

In summary, our analysis of a cohort database in a high burden country shows that compared to RH-FDC + Z, RHZE-FDC reduces the default rates by 14%, independently of other influencing individual or health service factors, which eventually have a stronger effect.

## Additional file

Additional file 1: Table S1. Individual risk factors for TREATMENT DEFAULT among pulmonary tuberculosis patients from October 2009 to September 2010. **Table S2.** Health care facility risk factors for TREATMENT DEFAULT among pulmonary tuberculosis patients from October 2009 to September 2010. **Table S3.** Individual risk factors for SECOND MONTH SMEAR NEGATIVATION among pulmonary tuberculosis patients from October 2009 to September 2010. **Table S4.** Health care facility risk factors for SECOND MONTH SMEAR NEGATIVATION among pulmonary tuberculosis patients from October 2009 to September 2010.
