# Supplementary material for: Effectiveness of RHZE-FDC (fixed-dose combination) compared to RH-FDC + Z for tuberculosis treatment in Brazil: a cohort study
Source: BMC Infect Dis. 2015 Feb 21;15:81. doi: 10.1186/s12879-015-0820-4 (PMC4352268; doi:10.1186/s12879-015-0820-4)
Supplement: Additional file 1: Table S1. — Individual risk factors for TREATMENT DEFAULT among pulmonary tuberculosis patients from October 2009 to September 2010. Table S2. Health care facility risk factors for TREATMENT DEFAULT among pulmonary tuberculosis patients from October 2009 to September 2010. Table S3. Individual risk factors for SECOND MONTH SMEAR NEGATIVATION among pulmonary tuberculosis patients from October 2009 to September 2010. Table S4. Health care facility risk factors for SECOND MONTH SMEAR NEGATIVATION among pulmonary tuberculosis patients from October 2009 to September 2010. [file 12879_2015_820_MOESM1_ESM.docx]

**Additional file 1**

Table S1 – Individual risk factors for TREATMENT DEFAULT among pulmonary tuberculosis patients from October 2009 to September 2010

| Characteristics | OR | 95% CI | _adj_OR | 95% CI |
| --- | --- | --- | --- | --- |
| FDC-RHZE |  |  |  |  |
| No | 1 |  | 1 |  |
| Yes | 0.811 | 0.723 - 0.910 | 0.897 | 0.728 - 1.106 |
| Age |  |  |  |  |
| < 38 years | 1 |  | 1 |  |
| ≥38 years | 0.620 | 0.555 - 0.692 | **0.599** | **0.484 - 0.742** |
| Sex |  |  |  |  |
| Feminine | 1 |  |  |  |
| Masculine | 1.501 | 1.334 - 1.689 |  |  |
| Illiteracy |  |  |  |  |
| No | 1 |  |  |  |
| Yes | 1.197 | 0.887 - 1.615 |  |  |
| Higher education occupation |  |  |  |  |
| No | 1 |  | 1 |  |
| Yes | 0.355 | 0.217 - 0.582 | **0.223** | **0.081 - 0.610** |
| Living in prisons |  |  |  |  |
| No | 1 |  |  |  |
| Yes | 0.967 | 0.812 - 1.152 |  |  |
| Alcohol use |  |  |  |  |
| No | 1 |  | 1 |  |
| Yes | 1.555 | 1.349 - 1.793 | **1.767** | **1.377 - 2.268** |
| Diabetes |  |  |  |  |
| No | 1 |  |  |  |
| Yes | 0.579 | 0.450 - 0.746 |  |  |
| Mental disorder |  |  |  |  |
| No | 1 |  |  |  |
| Yes | 0.668 | 0.436 - 1.023 |  |  |
| Other co-morbidities |  |  |  |  |
| No | 1 |  |  |  |
| Yes | 1.223 | 1.052 - 1.422 |  |  |
| DOT |  |  |  |  |
| No | 1 |  |  |  |
| Yes | 1.063 | 0.938 - 1.205 |  |  |
| HIV status |  |  |  |  |
| Negative | 1 |  | 1 |  |
| Positive | 1.725 | 1.426 - 2.087 | **1.869** | **1.499 - 2.331** |

FDC=fixed-dose combination; R=rifampin; H=isoniazid; Z=pyrazinamide; E=ethambutol, DOT=directly observed treatment; OR=odds ratio; _adj_OR=adjusted odds ration; CI=confidence interval

Table S2– Health care facility risk factors for TREATMENT DEFAULT among pulmonary tuberculosis patients from October 2009 to September 2010

| Characteristics | % | OR | 95% CI | _adj_OR | 95% CI |
| --- | --- | --- | --- | --- | --- |
| FDC-RHZE |  |  |  |  |  |
| No | 66.4 | 1 |  | 1 |  |
| Yes | 33.6 | 0.811 | 0.723 - 0.910 | **0.819** | **0.728 - 0.923** |
| The unit assists over 125 cases yearly | | | |  |  |
| No | 50.1 | 1 |  |  |  |
| Yes | 49.9 | 0.997 | 0.897 - 1.108 |  |  |
| The unit offers DOT | | |  |  |  |
| No | 18.5 | 1 |  | 1 |  |
| Yes | 81.5 | 0.946 | 0.826 - 1.082 | 0.979 | 0.850 - 1.128 |
| The unit requests follow up smears | | | | |  |
| No | 21.7 | 1 |  | 1 |  |
| Yes | 78.3 | 0.914 | 0.805 - 1.037 | 0.947 | 0.826 - 1.087 |
| The unit has a laboratory facility | | | |  |  |
| No | 65.2 | 1 |  | 1 |  |
| Yes | 34.8 | 0.904 | 0.808 - 1.012 | **0.892** | **0.796 - 1.001** |
| The unit has a complete team with physician, nurse and social worker | | | | | |
| No | 36.2 | 1 |  |  |  |
| Yes | 63.8 | 1.355 | 0.817 - 1.519 |  |  |
| The unit has a Social Service | | | |  |  |
| No | 36.2 | 1 |  |  |  |
| Yes | 63.8 | 1.224 | 0.756 - 1.914 |  |  |
| The unit has community health agents | | | |  |  |
| No | 50.4 | 1 |  |  |  |
| Yes | 49.6 | 1.917 | 0.778 - 2.139 |  |  |
| The unit has the Family Health Program | | | | |  |
| No | 56.1 | 1 |  |  |  |
| Yes | 43.9 | 1.688 | 0.987 - 1.878 |  |  |
| The unit has training activities | | | |  |  |
| No | 88.1 | 1 |  |  |  |
| Yes | 11.9 | 0.945 | 0.801 - 1.115 |  |  |
| The unit has primary care | | | |  |  |
| No | 44.1 | 1 |  |  |  |
| Yes | 55.9 | 1.553 | 0.524 - 1.734 |  |  |
| The unit has exclusively primary care | | | | |  |
| No | 84.4 | 1 |  |  |  |
| Yes | 15.6 | 1.404 | 0.887 - 1.828 |  |  |

FDC=fixed-dose combination; R=rifampin; H=isoniazid; Z=pyrazinamide; E=ethambutol, DOT=directly observed treatment; OR=odds ratio; _adj_OR=adjusted odds ration; CI=confidence interval

Table S3 - Individual risk factors for SECOND MONTH SMEAR NEGATIVATION among pulmonary tuberculosis patients from October 2009 to September 2010

| Characteristics | % | OR | 95% CI | _adj_OR | 95% CI |
| --- | --- | --- | --- | --- | --- |
| FDC-RHZE |  |  |  |  |  |
| No | 66.4 | 1 |  | 1 |  |
| Yes | 33.6 | 1.229 | 1.047 - 1.441 | **1.185** | **1.006 - 1.395** |
| Age |  |  |  |  |  |
| < 38 years | 52.3 | 1 |  |  |  |
| ≥38 years | 47.8 | 0.874 | 0.745 - 1.025 |  |  |
| Sex |  |  |  |  |  |
| Feminine | 34.8 | 1 |  |  |  |
| Masculine | 65.2 | 1.027 | 0.871 - 1.212 |  |  |
| Illiteracy |  |  |  |  |  |
| No | 96.4 | 1 |  |  |  |
| Yes | 3.7 | 1.179 | 0.754 - 1.841 |  |  |
| Higher education occupation |  |  |  |  |  |
| No | 96.4 | 1 |  |  |  |
| Yes | 3.7 | 1.864 | 0.888 - 3.908 |  |  |
| Living in prisons |  |  |  |  |  |
| No | 91.8 | 1 |  |  |  |
| Yes | 8.2 | 0.903 | 0.687 - 1.187 |  |  |
| Alcohol use |  |  |  |  |  |
| No | 81.8 | 1 |  |  |  |
| Yes | 18.2 | 0.802 | 0.645 - 0.996 |  |  |
| Diabetes |  |  |  |  |  |
| No | 92.4 | 1 |  |  |  |
| Yes | 7.6 | 0.946 | 0.705 - 1.270 |  |  |
| Mental disorder |  |  |  |  |  |
| No | 97.8 | 1 |  |  |  |
| Yes | 2.2 | 0.93 | 0.526 - 1.645 |  |  |
| Other co-morbidities |  |  |  |  |  |
| No | 81.3 | 1 |  |  |  |
| Yes | 18.7 | 0.798 | 0.630 - 1.025 |  |  |
| DOT |  |  |  |  |  |
| No | 75.9 | 1 |  | 1 |  |
| Yes | 24.1 | 1.45 | 1.208 - 1.739 | **1.418** | **1.181 - 1.704** |
| HIV status |  |  |  |  |  |
| Negative | 74.7 | 1 |  |  |  |
| Positive | 25.34 | 0.865 | 0.622 - 1.204 |  |  |

FDC=fixed-dose combination; R=rifampin; H=isoniazid; Z=pyrazinamide; E=ethambutol, DOT=directly observed treatment; OR=odds ratio; _adj_OR=adjusted odds ration; CI=confidence interval

Table S4 - Health care facility risk factors for SECOND MONTH SMEAR NEGATIVATION among pulmonary tuberculosis patients from October 2009 to September 2010

| Characteristics | % | OR | 95% CI | _adj_OR | 95% CI |
| --- | --- | --- | --- | --- | --- |
| FDC-RHZE |  |  |  |  |  |
| No | 66.4 | 1 |  | 1 |  |
| Yes | 33.6 | 1.229 | 1.047 - 1.441 | 1.162 | 0.987 - 1.366 |
| The unit assists over 125 cases yearly | | | |  |  |
| No | 50.1 | 1 |  | 1 |  |
| Yes | 49.9 | 0.782 | 0.663 - 0.922 | **0.756** | **0.638 - 0.895** |
| The unit offers DOT | |  |  |  |  |
| No | 18.5 | 1 |  | 1 |  |
| Yes | 81.5 | 1.423 | 1.126 - 1.794 | **1.364** | **1.073 - 1.733** |
| The unit requests follow up smears | | | | |  |
| No | 21.7 | 1 |  |  |  |
| Yes | 78.3 | … | … |  |  |
| The unit has a laboratory facility | | | |  |  |
| No | 65.2 | 1 |  |  |  |
| Yes | 34.8 | 0.998 | 0.841 - 1.185 |  |  |
| The unit has a complete team with physician, nurse and social worker | | | | | |
| No | 36.2 | 1 |  |  |  |
| Yes | 63.8 | 0.991 | 0.844 - 1.163 |  |  |
| The unit has a Social Service | | | |  |  |
| No | 36.2 | 1 |  |  |  |
| Yes | 63.8 | 0.991 | 0.844 - 1.163 |  |  |
| The unit has community health agents | | | |  |  |
| No | 50.4 | 1 |  |  |  |
| Yes | 49.6 | 1.027 | 0.867 - 1.216 |  |  |
| The unit has the Family Health Program | | | |  |  |
| No | 56.1 | 1 |  |  |  |
| Yes | 43.9 | 1.119 | 0.944 - 1.326 |  |  |
| The unit has training activities | | | |  |  |
| No | 88.1 | 1 |  |  |  |
| Yes | 11.9 | 1.264 | 0.665 - 2.402 |  |  |
| The unit has primary care | | | |  |  |
| No | 44.1 | 1 |  |  |  |
| Yes | 55.9 | 1.115 | 0.945 - 1.315 |  |  |
| The unit has exclusively primary care | | | |  |  |
| No | 84.4 | 1 |  | 1 |  |
| Yes | 15.6 | 1.289 | 1.065 - 1.559 | **1.3** | **1.070 - 1.980** |

FDC=fixed-dose combination; R=rifampin; H=isoniazid; Z=pyrazinamide; E=ethambutol, DOT=directly observed treatment; OR=odds ratio; _adj_OR=adjusted odds ration; CI=confidence interval
